# Weighted pseudo-values for partly unobserved group membership in paediatric stem cell transplantation studies

**DOI:** 10.1177/09622802211041756

**Published:** 2021-11-23

**Authors:** Martina Mittlböck, Ulrike Pötschger, Harald Heinzl

**Affiliations:** 1 Center for Medical Statistics, Informatics, and Intelligent Systems, 27271 Medical University of Vienna, Austria; 2 Children’s Cancer Research Institute, 535811 Vienna, Austria

**Keywords:** Cumulative hazard ratio, non-proportional hazards, selection bias, immortal time bias, generalised pseudo values, binary time-dependent covariate

## Abstract

Generalised pseudo-values have been suggested to evaluate the impact of allogeneic stem cell transplantation on childhood leukaemia. The approach compares long-term survival of two cohorts defined by the availability or non-availability of suitable donors for stem cell transplantation. A patient's cohort membership becomes known only after completed donor search with or without an identified donor. If a patient suffers an event during donor search, stem cell transplantation will no longer be indicated. In such a case, donor search will be ceased and cohort membership will remain unknown. The generalised pseudo-values approach considers donor identification as binary time-dependent covariate and uses inverse-probability-of-censoring weighting to adjust for non-identified donors. The approach leads to time-consuming computations due to multiple redefinitions of the risk set for pseudo-value calculation and an explicit adjustment for waiting-time bias. Here, the problem is looked at from a different angle. By considering the probability that a donor would have been identified after ceasing of donor search, weights for common pseudo-values are defined. This leads to a faster alternative approach as only a single risk set is necessary. Extensive computer simulations show that both, the generalised and the new weighted pseudo-values approach, provide approximately unbiased estimates. Confidence interval coverage is satisfactory for typical clinical scenarios. In situations, where donor identification takes considerably longer than usual, the weighted pseudo-values approach is preferable. Both approaches complement each other as they have different potential in addressing further aspects of the underlying medical question.

## 1 Background

Childhood leukaemia studies that compare allogeneic stem cell transplantation (SCT) with conventional chemotherapy usually exhibit specific features that result in statistically challenging data analyses. After being considered eligible for SCT, a patient is included in the study, conventional chemotherapy is administered, and donor search is started in international donor registries if no suitable related donor has been identified. These registries contain pre-typed blood sample data of volunteers willing to become stem cell donors. If a potential donor is pre-selected and his/her willingness could be re-confirmed, then a new blood sample will be exactly typed. If a match is achieved, chemotherapy can be stopped and SCT can be performed. Chemotherapy is the sole treatment for those patients, for whom no matched donor can be identified during the search period. According to their donor availability status, patients belong to either of two cohorts, that is, with and without available donor.

It may happen that a patient suffers death, relapse, or other primary disease-specific problems during the donor search period. Consequently, the indication for SCT will be lost, donor search will be ceased, and donor availability status will remain unknown for this patient. Note that donor availability status can, nonetheless, be considered as an external covariate as it exists beyond patient survival or disease progression.^1^ After successful donor identification, SCT will be performed unless contraindications prevent it. Therefore, in order to avoid selection bias, it is crucial that statistical approaches consider donor identification rather than SCT applied.

Ideally, the so-called genetic randomisation approach would be used to evaluate SCT trials.^2–4^ This approach mimics the intention-to-treat principle of randomised trials by comparing the long-term survival of patients with available donor (SCT possible) and without available donor (SCT impossible). Unfortunately, missing donor availability status makes it often impossible to apply genetic randomisation and alternative approaches have to be considered.

The prevailing Cox model with a binary time-dependent covariate is hampered by non-proportional hazards. The benefits of an SCT are expected to fully unfold on the long run; on the short run, an increase in treatment-related events is commonly observed. As the Cox model considers relative instantaneous risks, it cannot be used to assess cumulative survival effects in a non-proportional hazard situation.^5,6^ Landmark analysis and dynamic prediction by landmarking are other popular approaches;^7–10^ however, their results can be affected by the arbitrarily chosen landmark time. Pötschger et al.^11^ discuss all these and other approaches in more detail and suggest a new approach based on so-called generalised pseudo-values (GPVs) which considers long-term survival, mimics the intention-to-treat principle, yet overcomes the missing donor availability and the non-proportional hazards problem.

Unfortunately, the practical application of GPVs is computationally intensive and time-consuming. This is, among others, especially problematic during study design, when the required sample size or statistical power has to be assessed by means of computer simulation. Furthermore, suboptimal confidence interval coverage rates were observed in certain cases, and an ad-hoc parametric bootstrap procedure was developed to overcome this issue.^11^ Meanwhile, a programming error in the original implementation of the procedure was discovered. The error has, by coincidence, resulted in reasonable confidence intervals for the cases considered^11^; however, after error recovery, it became apparent that the intervals are in fact too wide leading to unacceptably high coverage rates. As no workable alternative solution is available, the ad-hoc parametric bootstrap procedure will not be pursued any further for the GPV approach.

Here, the new approach of weighted pseudo-values (WPVs) is proposed as alternative to GPVs; both estimating cohort survival probabilities at a pre-specified time point and the associated cumulative hazard ratio . WPVs are based on an individually adapted probability weighting of common pseudo-values and can be efficiently computed in a single step. In the following, GPVs and WPVs will be introduced in the section ‘Methods’. They will be compared by means of a simulation study in the section ‘Results’. Further issues and additional aspects will be summarised and discussed in the section ‘Discussion’.

## 2 Methods

In the following, the term survival is linked to the outcome-related events of interest, like death, or a composite endpoint that additionally includes relapse or other disease-related events. Furthermore, the term donor search-ceasing events comprise all events that permanently contraindicate SCT and thus lead to stop of donor search. Note that donor search-ceasing events do not have to be identical with outcome-related events. For example, if overall survival is studied, then death of patient will cease donor search as well as define the outcome of interest, whereas a relapse will only cease donor search. Since donor availability is an external covariate, it is independent of these donor search-ceasing events.

The survival probability at time of patients with a donor available, , is to be compared with the survival probability of those without an available donor, . Here, is a pre-specified time point used to indicate long-term survival or cure of the patients.

**Figure 1.**
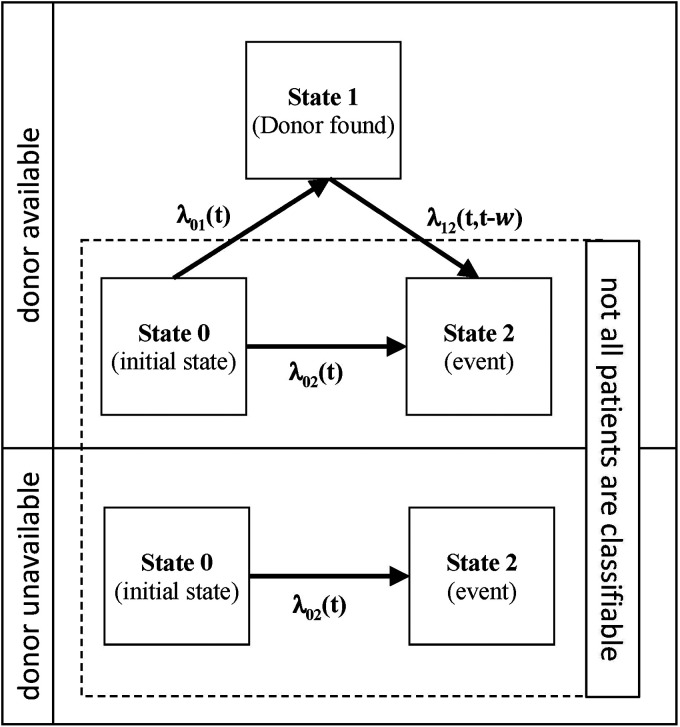
Stochastic processes of the two identical populations that only differ with regard to their donor availability status; , and denote hazard functions, and *w* is the time of transition to state 1. In the donor available group, all patients leave state 0 either to state 1 or state 2 by no later than .

The maximum donor search time is used to determine the donor availability status (see Figure 1). That is, a donor is considered available/not available, if it can/cannot be identified up to , respectively. If a patient without identified donor suffers a donor search-ceasing event (or is censored) before , then the donor availability status will remain unknown for this patient.

Define the random variable *W*, , as waiting time in the initial state 0 until a donor is identified and the patient moves to state 1. Let and denote the hazard function and the density of the distribution of *W*, respectively. Then the survival probability at for patients with an available donor is

$$S_{1}(t^{*})=\int_{0}^{t^{*}}\;f_{01}(w)S_{1}(t^{*}|w)dw,$$

where denotes survival in a population with donor available at a fixed waiting time . The hazard function describes the transition from state 1 to the event state 2; it can depend on the time *t* since the patient entered the study (in initial state 0) as well as the time since a donor was identified and the patient moved to state 1.

The survival probability at for patients without donor available is

$$S_{0}(t^{*})=\exp\;\left[-\left\{\int_{0}^{t^{*}}\lambda_{02}(v)dv\right\}\right]$$

where is the hazard function of transition from initial state 0 to the final state 2 (Figure 1).

Note that the hazard for the direct transition from the initial state 0 to the event state 2 is equal for both populations up to ; thereafter there will be, by definition, no patients anymore in state 0 of the donor available population.

The cHR at time t* for the comparison of patients with and without an available donor is .

### 2.1 Common pseudo-values

Given *n* patients, the common pseudo-value at time for the *i*th patient is

(1) $${\hat{V}}_{i}(t^{*})=n\hat{S}(t^{*})-(n-1){\hat{S}}^{-i}(t^{*}),$$,

where denotes the Kaplan-Meier estimate at and denotes the Kaplan-Meier estimate at without the *i*th patient.^12^

As common pseudo-values are asymptotically conditionally unbiased given covariates, they can be used as a response variable in a regression model.^13,14^ However, i.i.d. observations and independent right censoring have to be assumed; and the risk set at time has to be sufficiently large.

### 2.2 Generalised pseudo-values

Assume that for the first *m* out of *n* patients a transition to state 1 has been observed, and denote the observed transition times . The GPV approach of Pötschger et al.^11^ now defines GPVs, .

Here , , are common pseudo-values with denoting the Kaplan-Meier estimate at for the risk of a direct transition from state 0 to state 2; where patients with an available donor are censored at their observed transition times .

The remaining *m* GPVs are , with , . has just been introduced above; and is the Kaplan-Meier estimate at based on all patients still at risk at .

Finally, weights have to be defined for . Trivially, for . In contrast, are needed to adjust for unobserved 0 → 1 transitions due to donor search-ceasing events and censoring. This is achieved by inverse probability weighting with , the Kaplan-Meier estimate of all *n* patients where both, donor search-ceasing events and censoring, are considered as events; whereas observed 0 → 1 transitions are considered as censored at . That is, , ; note that .

Now, and are consistent estimators of and , respectively.

GPVs can be used as response variable in a weighted generalised linear model with log-minus-log link function , a normal response probability distribution, and a robust sandwich variance estimator. Let with for and otherwise. With the linear predictor , the parameter estimates correspond to and , so that and are the estimates for and , respectively. Finally, provides a consistent estimate of the cHR at time t* for the comparison of patients with and without an available donor.

### 2.3 Weighted pseudo-values

The novel WPV approach proportionally allocates a patient with uncertain group membership into the two groups with and without donor according to its probability that a donor might still be found until . There are patients (Table 1); for notational convenience assume that the first patients are those where it is known that a donor is unavailable (i.e. none has been identified until ), the next *m* patients are those with an identified donor; and the remaining patients are those where donor search was ceased and group membership is unknown.

**Table 1.** Weights of the WPV and the GPV approach.

Observed patients	WPV weights for the estimation of		GPV weights for the estimation of	
				
Without donor	1	0	1	–
With donor	0	1	1	
Donor search ceased			1	–
Sum of weights per column				
Total sum of weights				

For the latter patients, let denote the probability that a donor will be identified until , given that no donor has been found until , the time when donor search was ceased. Now, , where is the probability that no donor has been found for the *i*th patient by , so that, consequently, is the probability of belonging to the group without donor (additional details are provided in Section A of Supplemental Materials). A natural estimator of is provided by ; where is the Kaplan-Meier estimate for time to donor identification. Note that here all donor search-ceasing events as well as censoring are considered as censoring events, which are non-informative with regard to time to donor identification.

Consequently, the probability of no donor available is estimated by , . Note that if – for some reason – a donor is found after donor search has already been ceased, then it would be sensible to include this knowledge by setting for the patient concerned.

Now, for all patients common pseudo-values , , (see equation (1)) are calculated.

The WPV approach defines a vector with of these common pseudo-values , where the pseudo-values of the patients of the group, , occur twice. The corresponding weight vector is with for , for , and for _._

Let indicate group membership with for and and otherwise. Then and are consistent estimators of and , respectively.^13,15,16^

The vector can be used as response variable in a weighted generalised linear model with log-minus-log link , a normal response probability distribution, and a robust sandwich variance estimator. Using the linear predictor , then, in analogy to the GPV approach, the parameter estimates correspond to and , respectively, and is a consistent estimator of the cHR at time for the comparison of patients with and without an available donor.

An overview of the weights of both approaches and their contribution to the estimation of and can be found in Table 1. A SAS macro for the WPV approach is available via GitHub.^17,18^

## 3 Results

### 3.1 Simulation studies

The two simulation studies of Pötschger et al.^11^ are used to enable a straightforward comparison of the GPV with the WPV approach. In short, survival times were generated using the inversion method,^19^ which was extended by using a parametric mixture cure model^20^ to allow for a plateau of the survival curve that represents cured patients. Uniform censoring times between 0 to 6 years and 0 to 11 years were superimposed in the simulation scenarios of simulation studies 1 and 2, respectively. For each scenario, sample sizes of 400 and 1000 were generated for 1000 simulation runs.

At first, the WPV approach is applied to the original simulated data sets of Pötschger et al.,^11^ then the WPV results are compared to the original GPV results (Figures 2 to 5). Results of a considerably extended version of simulation study 2 are provided in the Supplemental Materials (Section D, Figures S2 to S13).

**Figure 2.**
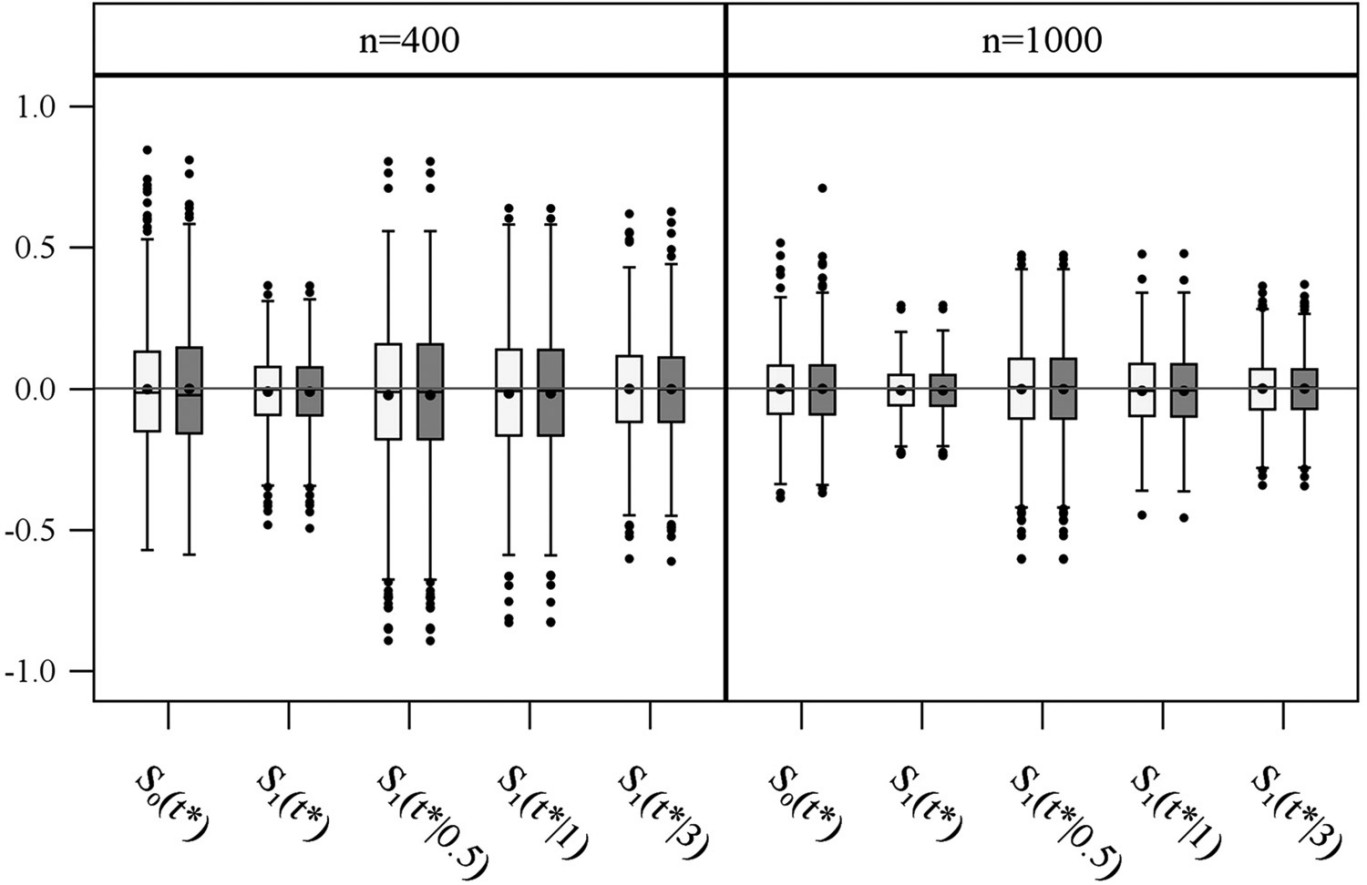
Bias distribution of log-minus-log survival probabilities estimated with the GPV (light grey) and WPV (dark grey) approach in simulation study 1 (1000 repetitions) for sample sizes of 400 and 1000. By definition, a donor can only be identified at 0.5, 1 or 3 years after start of donor search.

GPV: generalised pseudo-value; WPV: weighted pseudo-value.

**Figure 5.**
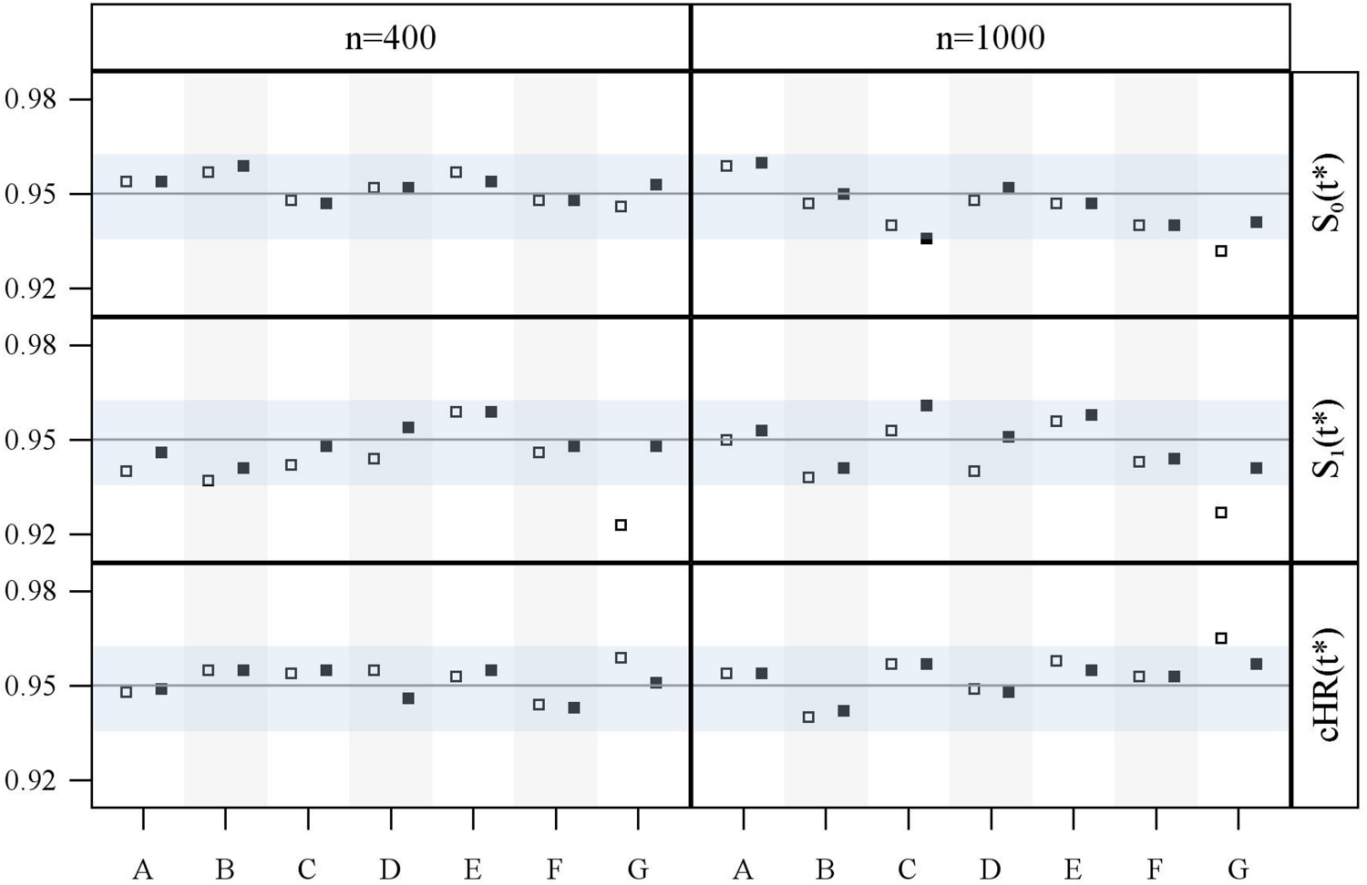
Observed coverage rates of 95% confidence intervals for survival probabilities and cumulative hazard ratios estimated with the GPV (square) and WPV (square-filled) approach for simulation study 2 (1000 repetitions) and sample sizes of 400 and 1000, for each of the seven scenarios A–G (see Figure S1 in Section C of Supplemental Materials).

GPV: generalised pseudo-value; WPV: weighted pseudo-value.

For the sake of convenience, the bias distributions (Figures 2, 4, and Supplemental Figures S2 to S7) are labelled , and , although they actually show the bias of the corresponding parameter estimates of the particular generalised linear models; that is, , and for the GPV, and , and for the WPV approach, respectively.

**Figure 4.**
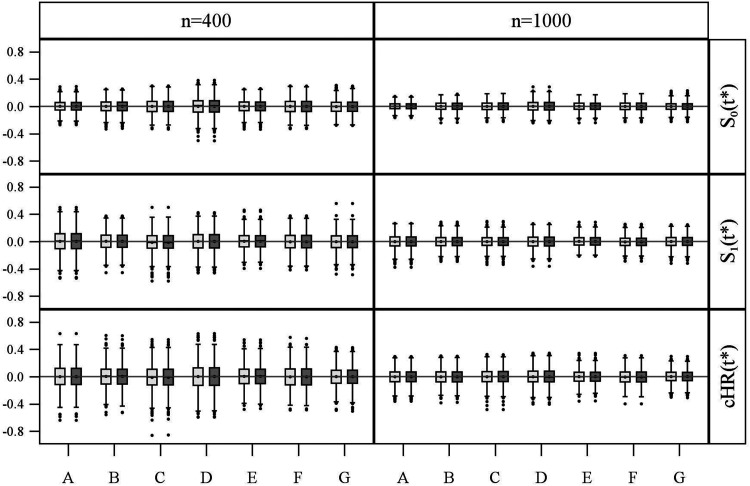
Bias distribution of log-minus-log survival probabilities and log-cumulative hazard ratios estimated with the GPV (light grey) and WPV (dark grey) approach in simulation study 2 (1000 repetitions) for sample sizes of 400 and 1000, for each of the seven scenarios A–G (see Figure S1 in Section C of Supplemental Materials).

GPV: generalised pseudo-value; WPV: weighted pseudo-value.

**Figure 3.**
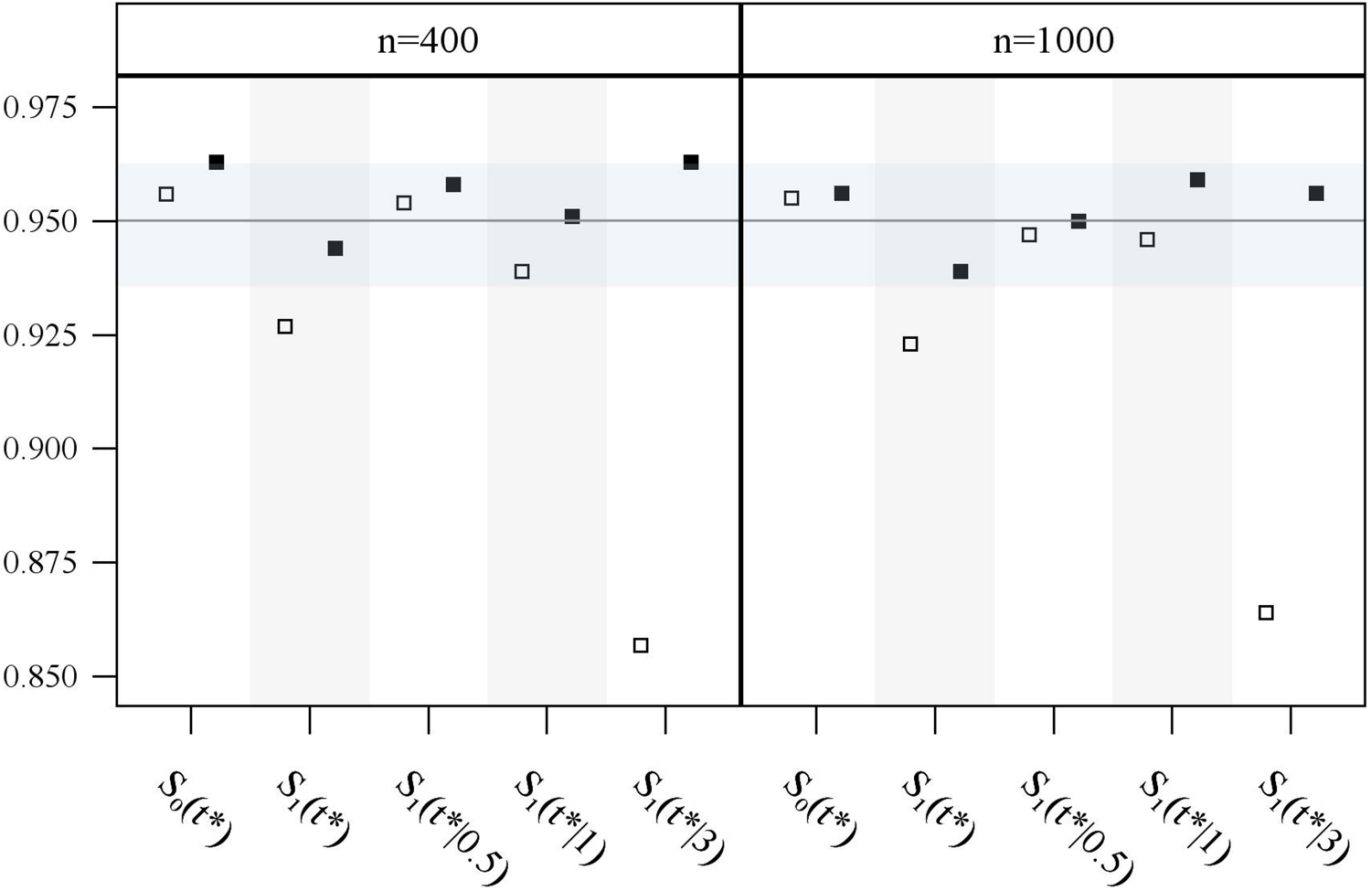
Observed coverage rates of 95% confidence intervals for survival probabilities estimated with the GPV (square) and WPV (square-filled) approach for simulation study 1 (1000 repetitions) and sample sizes of 400 and 1000. By definition, a donor can only be identified at 0.5, 1 or 3 years after start of donor search.

GPV: generalised pseudo-value; WPV: weighted pseudo-value.

The grey horizontal band in the confidence interval (CI) coverage plots (Figures 3, 5, and Supplemental Figures S8 to S13) marks the area between the 0.025 and the 0.975 quantile of a binomial distribution with parameters 1000 (number of trials, here a number of simulation runs) and 0.95 (success probability, here true confidence level ). That is, after 1000 simulation runs, the observed coverage rate of a CI with a true confidence level is contained in this band with a probability of 0.95 . Hence, an observed coverage rate below or above this band may be interpreted as indication that the considered CI actually is too liberal or too conservative, respectively.

Both approaches, GPV and WPV, can be implemented using standard routines available in the majority of statistical software packages; the following numerical results have been obtained with SAS software.^17^

#### Simulation study 1

This simulation study was performed to examine bias and 95% CI coverage rate of the survival probabilities of the two approaches in an extreme setting with discrete waiting times at either 0.5, 1 or 3 years. No donors are available for 25% of the patients. Donors are available for the remaining 75% so that always 25% belong to each of the three waiting times of 0.5, 1 and 3 years, respectively. The outcome is evaluated at 5 years .

As donor search-ceasing events or censoring prevent donor identification, available donors are identified on average only with a probability of 0.88, 0.74 and 0.28 at 0.5, 1 and 3 years, respectively. Consequently, only 22%, 18% and 7% of all patients (*m* in Table 1) will have donors identified at 0.5. 1 and 3 years, respectively, and the remaining 53% will have no identified donor (, see Table 1); remember that by definition there are 25% of patients in each of the four groups.

The GPV approach accounts for this missing observations at the three discrete donor identification times by weighting observed donor identifications to reconstruct the true waiting time distribution, leading to average estimated weights of 0.72, 0.86 and 2.26 for *w* = 0.5, 1 and 3, respectively.

In contrary, the WPV approach reduces the number of patients in the group of no identified donor and enriches the donor groups with waiting times at 0.5, 1 and 3 years according to their probability that a donor might be identified beyond patients' observed survival time. That is, the underlying group frequencies of 25% for all four subgroups are approximately restored. This enrichment of the three donor available groups to the cost of the no identified donor group exemplifies how the WPV approach prevents immortal time bias.

Figure 2 shows that both approaches result in unbiased parameter estimates for the no donor group as well as the donor available groups; either when donor availability is considered in general, or when survival is separately estimated for the given waiting times at 0.5, 1 and 3 years (see Supplemental Materials, Section B). Figure 3 gives coverages of 95% CIs for , , , and , respectively. The CI coverage rates for of the GPV approach are much too low as donor identification occurs late at 3 years; this feature also shows through in the general case . The novel and computationally easier WPV approach shows very good CI coverage rates in all situations.

#### Simulation study 2

This simulation study is performed to assess unbiasedness of the parameter estimates and CI coverage of both approaches in four realistic settings from paediatric oncology and three theoretical settings.^11^ The realistic situations represent the most common departures from proportional hazards: differences in long-term survival only (A), crossing survival curves (B, C), and a situation where only differences in short-term survival (D) are observed. The theoretical settings consider a proportional hazards model (E), a no-effect model (F), and a scenario with unrealistically long waiting times (G); see Figure S1 in Supplemental Materials, Section C. The proportions of available donors are 0.25 for scenario A, 0.4 for scenarios B–F and 0.45 for scenario G, respectively. The outcome is evaluated at 5 years , and the donor search period is set to 5 years , too.

Figure 4 shows that both approaches result in unbiased parameter estimates for all seven simulation scenarios A-G and the sample sizes and , respectively.

Coverage rates of 95% CIs are provided in Figure 5. Note that the CI for is the most important one as it corresponds to a statistical test of comparing both therapies at . For both approaches, the 95% CI of shows good coverage except for GPV in scenario G when *n* = 1000. This, however, seems to be a simulation artefact as the performance for *n* = 400 is adequate. WPV shows acceptable coverage in all situations for both, and the cumulative hazard ratio ; however, the coverage rates of GPV are only acceptable for scenarios A–F. Analogous to simulation study 1, GPV shows a too low coverage for when donor identification takes considerably longer (scenario G); this is accompanied by a slightly conservative coverage for when *n* = 1000.

Additional extensive simulation results can be found in Section D of Supplemental Materials, where the proportion of available donors and have been varied. Again, the general unbiasedness of both approaches turns out (Supplemental Figures S2 to S7).

The CI coverage rates for and in Supplemental Figures S8 to S13 confirm the results of Figure 5; both approaches perform well in scenarios A–F with realistic waiting time distribution, irrespective of donor availability proportion and -value. For scenario G, considerably low CI coverage rates for emerge again for the GPV approach, whereas the WPV approach shows adequate CI coverage (Supplemental Figures S8 to S13).

Both approaches show adequate CI coverage rates for in scenarios A–F. For the extreme scenario G, in particular, when , too high coverage rates may occur in both approaches (Supplemental Figures S8 to S13).

In summary, the good performance in nearly all investigated settings shows that the WPV approach does not depend on the proportional hazards assumption and that it is able to properly address immortal time bias.

### 3.2 Real data example: SCT in paediatric leukaemia patients

Below, the WPV result is compared to the results of a Cox model and the GPV approach using real data from an international study in 542 children with newly diagnosed Philadelphia chromosome-positive (PH+) acute lymphoblastic leukaemia.^11,21^ The study compared SCT to conventional chemotherapy; the primary endpoint was disease-free survival (DFS). For 325 of 542 patients, a donor could be identified, so that they could switch from chemotherapy to SCT after a median waiting time of 5.1 months. The remaining 217 patients were treated with chemotherapy only. Here we assume that donor identification leads to SCT in a timely manner. The Cox model with a binary time-dependent covariate (indicating SCT after donor identification) gives a hazard ratio (HR) of 0.91 in favour of SCT (95% CI: 0.72–1.14; *p* = 0.39).

**Figure 6.**
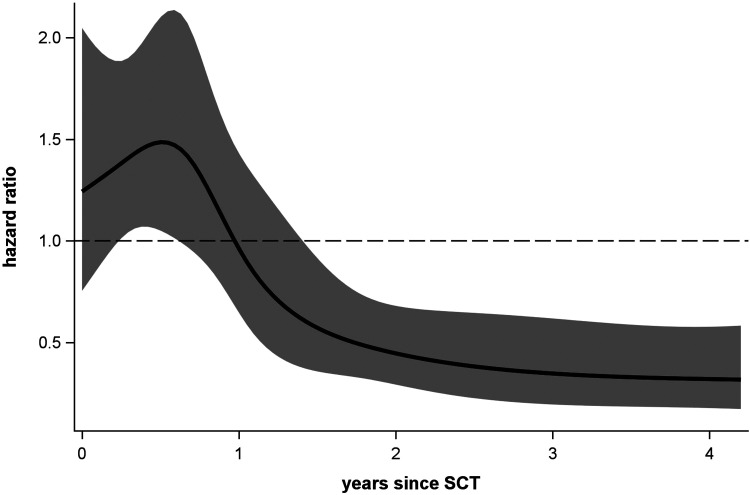
Instantaneous hazard ratio function and 95% pointwise confidence band for the PH+ acute lymphoblastic leukaemia dataset estimated by a restricted cubic spline function with 5 knots at 0.24, 0.68, 1.09, 1.86 and 4.2 years (corresponds to 5%, 27.5%, 50%, 72.5% and 95% of all outcome related events); a hazard ratio less than one favours stem cell transplantation (SCT).

This result is questionable as it assumes a constant SCT effect on DFS. The problem is exemplified in Figure 6 where the instantaneous HR after SCT is flexibly plotted over time.^22–24^ As expected, SCT initially results in an increased hazard (compared to conventional chemotherapy) with a maximum at about 0.5 years and decreasing risk thereafter. At about 1 year instantaneous hazards in both groups are equal (HR = 1), nevertheless, cumulated risks until 1 year are still to the disadvantage of SCT. However, it can neither be seen from the (non-significant) constant nor from the (significant) time-flexible HRs (Figure 6) whether SCT has a positive benefit–risk balance after all. Hence, since long-term survival is the crucial and almost exclusive interest in paediatric oncology, here assessed at 5 years after patients have entered the study, the Cox model with time-flexible hazard ratios will admittedly bring some additional insights, yet it cannot be considered the optimal statistical approach for treatment decisions.

The 5-year DFS estimates with and without donor are 41.7% (95% CI: 36.7%–46.5%) and 32.1% (25.4%–38.9%) for the GPV approach, and 41.7% (36.6%–46.7%) and 31.6% (24.6%–38.7%) for the WPV approach, respectively. The GPV approach yields a 5-year cHR of 0.77 favouring SCT (95% CI: 0.61–0.97; *p* = 0.027), the easier to calculate WPV approach gives a similar cHR of 0.76 (95% CI: 0.60–0.96; *p* = 0.021). Hence, it can be concluded that the long-term benefit of SCT outweighs its short-term risk (Figure 6).

For 325 patients, a donor is identified ( in Table 1). For 217 patients with no identified donor (, see Table 1), it is known that patients do not have a donor available. Now, with the WPV approach, it is straightforward to communicate that for the remaining *n_C_* = 172 patients it is expected that about 38 of them belong to the donor available group, and about 134 do not have a suitable donor available. Overall, it is expected that 67% of all patients have a suitable donor registered in the databases.

## 4 Discussion

The choice between two treatment strategies with respect to long-term survival is not straightforward, when the feasibility of one of the strategies emerges over time, non-proportional hazards are present, and there is a risk for immortal time bias. A typical example where all these issues co-occur are childhood leukaemia studies comparing allogeneic SCT with conventional chemotherapy.

It is expected that SCT causes a short-term increase in the hazard for overall or disease-free survival and yields a long-term benefit thereafter^3,25,26^ (also see Figure 6). Due to patient-specific reasons, donor search may be ceased and an available donor cannot be identified anymore. Therefore, in order to allow a fair comparison between SCT and the competing treatment strategy, the latent binary covariate donor availability has to be considered in statistical analysis.

The WPV approach uses common pseudo-values and attaches appropriate weights to them to reconstruct the cohorts with and without donor available (Figure 1); that is, the weighted partitioning of observations with undefined group membership is the key feature of the method. It is important to note, that the weights depend on the elapsed time of donor search. The more time has been elapsed, the less likely is the identification of an available donor.

In contrast, the GPV approach generalises common pseudo-values in various sophisticated ways to account for immortal time bias and related issues, and weights them thereafter to reconstruct the distribution of the time to donor identification, which is affected by donor search-ceasing events and censoring. Hence, the generalisation of the pseudo-values is the key feature of this method.

Both approaches are approximately unbiased and show reasonable CI coverage in scenarios with waiting time distributions that are typical for stem cell transplantation studies with non-related donors. However, confidence interval coverage, in particular of the GPV approach, may be negatively affected in situations where it takes much longer than usual to find suitable donors.

With regard to ease of software implementation and computational speed, WPV clearly outperforms GPV. The former approach requires the computation of common pseudo-values only, whereas the latter approach requires different rounds of computations with different starting times and patient risk sets. In the considered simulation scenarios of Section D of Supplemental Materials, the median WPV runtime was at least 40 times to more than 90 times faster than the corresponding median GPV runtime (see Figure S14 in Section E of Supplemental Materials). The WPV runtime advantage over GPV increases with the proportion of identified donors as more computationally time-consuming -values have to be calculated. The smaller runtime advantage in scenario G compared to scenarios A–F confirms that the proportion of identified rather than available donors is crucial (Supplemental Figure S14), as the considerably longer waiting times of scenario G lead to less identified donors and, consequently, less -calculations in the GPV approach.

When it comes to interpretation, both approaches provide the same primary message in terms of long-term survival estimation and group difference testing; however, both approaches may give different additional insights. Consider the real data example above (PH+ acute lymphoblastic leukaemia patients); here the WPV approach enables to directly conclude that an extremely fast donor search might have identified up to about 38 additional donors. Nonetheless, there are no donors available for around 33% of all patients; a number that can only be reduced by increasing the number of potential stem cell donors. In contrast, the GPV approach might be used to investigate other (artificial) distributions of the time to donor identification by adapting the inverse probability weights. By doing so, effects of accelerated or deferred donor identification on patients' survival could be directly studied.

Finally, consider the completely different setting of a diagnostic study with survival time outcome. To enable the computation of common diagnostic measures (like sensitivity, specificity) at a pre-specified time point, Antolini and Valsecchi^27^ suggested to impute the disease status (case or control) of censored subjects according to the probability that the subject is a case or a control. The weights of this “direct estimation by imputation”—approach share the same underlying idea as the WPV-weights.

## Conclusion

When donor availability status is unknown due to donor search-ceasing events or censoring, then both, the WPV and the GPV approach, are able to successfully deal with the time-dependent nature of donor identification and partially unobserved donor availability, thereby mimicking the genetic randomization approach for SCT trials in paediatric oncology.^3^ From a practical point of view, the WPV approach is computationally faster and easier to implement than the GPV approach. Previous SCT studies that have been analysed within the Cox model framework may be reanalysed with the new approach to reassess treatment effects and resulting treatment recommendations.

## Supplementary Material

Supplementary material

Supplementary material
