# Supplementary material for: Weighted pseudo-values for partly unobserved group membership in paediatric stem cell transplantation studies
Source: Stat Methods Med Res. 2021 Nov 23;31(1):76–86. doi: 10.1177/09622802211041756 (PMC8721556; doi:10.1177/09622802211041756)
Supplement: Supplementary material [file sj-pdf-1-smm-10.1177_09622802211041756.pdf]

Supplemental material for

Weighted pseudo-values for partly unobserved group membership in paediatric stem cell transplantation studies

Martina Mittlböck[1], Ulrike Pötschger[2], Harald Heinzl[1]

1 Center for Medical Statistics, Informatics, and Intelligent Systems, Medical University of Vienna, Vienna, Austria

2 Children's Cancer Research Institute, Vienna, Austria

Section A: Calculation of weights for the WPV approach

$S_D\left(t^{search}\right)$ is the probability that no donor is available at all, and $S_{01}\left(t_i\right)=\exp\left[-\int_0^{t_i}\lambda_{01}\left(v\right)dv\right]$ is the probability that an available donor has not been found until t_i. Then $S_D\left(t_i\right)=S_D\left(t^{search}\right)+\left(1-S_D\left(t^{search}\right)\right)S_{01}\left(t_i\right)$ is the probability that no donor has been found for the *i*-th patient by t_i, for $0\le t_i\le t^{search}$. Hence, $S_D\left(t_i\right)$ follows a mixture model for the dichotomous donor availability and the continuous time to donor-identification which is in analogy to the mixture cure model of Sposto [1], with “no donor available” corresponding to the meaning of “cure”.

Now, the probability that a donor will be identified until t^{search}, given that no donor has been found until t_i, is

$$\begin{aligned}\kappa_i &= \frac{S_D\left(t_i\right)-S_D\left(t^{search}\right)}{S_D\left(t_i\right)}\\ &= \frac{\left[S_D\left(t^{search}\right)+\left(1-S_D\left(t^{search}\right)\right)S_{01}\left(t_i\right)\right]-S_D\left(t^{search}\right)}{S_D\left(t^{search}\right)+\left(1-S_D\left(t^{search}\right)\right)S_{01}\left(t_i\right)}\\ &= \frac{\left(1-S_D\left(t^{search}\right)\right)S_{01}\left(t_i\right)}{S_D\left(t^{search}\right)+\left(1-S_D\left(t^{search}\right)\right)S_{01}\left(t_i\right)}\end{aligned}$$

Denote the Kaplan-Meier estimate for time to donor identification by $\hat{S}_D\left(t\right)$, where all donor search ceasing events as well as censoring are considered as censoring events. Then κ_i can be estimated by $\hat{\kappa}_i=\frac{\hat{S}_D\left(t_i\right)-\hat{S}_D\left(t^{search}\right)}{\hat{S}_D\left(t_i\right)}$. It is interesting to note that if a donor search ceasing event or censoring occurs after the longest observed donor identification time but before t^{search}, then $\hat{\kappa}_i=0$ and the corresponding patient will be allocated at 100 % to the no donor available group. Hence, any t^{search}-value between the longest observed donor identification time and $t*$ will yield the same WPV estimates.

Section B: WPV-weights for simulation scenario 1 with a discrete waiting time distribution

In simulation study 1, two WPV models have been considered. The first model estimates $S_0(t^*)$ and $S_1(t^*)$, whereas the second one estimates $S_0(t^*)$, $S_1(t^*|0.5)$, $S_1(t^*|1)$, and $S_1(t^*|3)$.

WPV model 1

This WPV model has already been described in the main paper. We repeat it here for the sake of convenience.

$\hat{\mathbf{V}}_\mathbf{D} = \left(\hat{V}_1(t^*),...,\hat{V}_n(t^*),\hat{V}_{n_U+m+1}(t^*),...,\hat{V}_n(t^*)\right)'$ is a vector with $n+n_C = n_U + m + 2n_C$ common pseudo-values, where the pseudo-values of the patients of the n_C group, $\hat{V}_{n_U+m+1}(t^*),...,\hat{V}_n(t^*)$, occur twice. The corresponding weight vector is $\hat{\boldsymbol{\gamma}}_\mathbf{D} = \left(\hat{\gamma}_{1,D},\ldots,\hat{\gamma}_{n+n_C,D}\right)'$ with $\hat{\gamma}_{i,D} = 1$ for $i = 1\ldots n_U + m$, $\hat{\gamma}_{i,D} = 1 - \hat{\kappa}_{i-(n_U+m)}$ for $i = n_U + m + 1\ldots n$, and $\hat{\gamma}_{i,D} = \hat{\kappa}_{i-n}$ for $i = n+1\ldots n+n_C$. The vector $\mathbf{x}_\mathbf{D} = \left(x_{1,D},\ldots,x_{n+n_C,D}\right)'$ indicate group membership with $x_{i,D} = 0$ for $i = 1\ldots n_U$ and $i = n_U + m + 1\ldots n$, and $x_{i,D} = 1$ otherwise. Finally, the vector $\hat{\mathbf{V}}_\mathbf{D}$ is used as response variable in a weighted generalised linear model with linear predictor $\beta_{0,D} + \beta_{1,D}x_{i,D}$. The estimated regression parameters provide the basis for estimates of $S_0(t^*)$ and $S_1(t^*)$; here at $t^* = 5$ years.

WPV model 2

Since donors can only be identified after waiting times of 0.5, 1, and 3 years, t^{search} equals 3 years by definition. Now if donor search is ceased at t_i, then

- $\hat{S}_D(t_i) = 1$ for $0 \le t_i < 0.5$
- $\hat{S}_D(t_i) = \hat{S}_D(0.5)$ for $0.5 \le t_i < 1$
- $\hat{S}_D(t_i) = \hat{S}_D(1)$ for $1 \le t_i < 3$

Hence, $\hat{\kappa}_i$ will be either $1-\hat{S}_D(3)$, $\frac{\hat{S}_D(0.5)-\hat{S}_D(3)}{\hat{S}_D(0.5)}$, or $\frac{\hat{S}_D(1)-\hat{S}_D(3)}{\hat{S}_D(1)}$, and $1-\hat{\kappa}_i$ will be either $\hat{S}_D(3)$, $\frac{\hat{S}_D(3)}{\hat{S}_D(0.5)}$, or $\frac{\hat{S}_D(3)}{\hat{S}_D(1)}$, respectively.

Having this in mind, we can now consider the second WPV model, where survival in the donor available group is separately estimated depending on the time, when the donor may be identified, denoted by $S_1(t^*|0.5)$, $S_1(t^*|1)$, and $S_1(t^*|3)$, respectively. The weights are shown in Table S1.

Table S1: WPV-weighting scheme for the second model of simulation study 1. It can be realised by appropriately defining the weight vector and the three group membership vectors.

observed patients		$S_0(t^*)$	$S_1(t^*\|0.5)$	$S_1(t^*\|1)$	$S_1(t^*\|3)$
without donor	n_U	1	0	0	0
with donor identified	m				
at $t_i=0.5$		0	1	0	0
$t_i=1$		0	0	1	0
$t_i=3$		0	0	0	1
donor search ceased	n_C				
between $0\le t_i<0.5$		$\hat{S}_D(3)$	$1-\hat{S}_D(0.5)$	$\hat{S}_D(0.5)-\hat{S}_D(1)$	$\hat{S}_D(1)-\hat{S}_D(3)$
$0.5\le t_i<1$		$\frac{\hat{S}_D(3)}{\hat{S}_D(0.5)}$	0	$\frac{\hat{S}_D(0.5)-\hat{S}_D(1)}{\hat{S}_D(0.5)}$	$\frac{\hat{S}_D(1)-\hat{S}_D(3)}{\hat{S}_D(0.5)}$
$1\le t_i<3$		$\frac{\hat{S}_D(3)}{\hat{S}_D(1)}$	0	0	$\frac{\hat{S}_D(1)-\hat{S}_D(3)}{\hat{S}_D(1)}$

Here, $\hat{\mathbf{V}}_{\mathbf{D}} = \left(\hat{V}_1(t^*),\dots,\hat{V}_n(t^*),\hat{V}_{n_U+m+1}(t^*),\dots,\hat{V}_n(t^*),\hat{V}_{n_U+m+1}(t^*),\dots,\hat{V}_n(t^*),\hat{V}_{n_U+m+1}(t^*),\dots,\hat{V}_n(t^*)\right)'$ is a vector with $n+3n_C = n_U + m + 4n_C$ of common pseudo-values, where the pseudo-values of the patients of the n_C group, $\hat{V}_{n_U+m+1}(t^*),\dots,\hat{V}_n(t^*)$, occur in quadruple.

The corresponding weight vector is $\hat{\boldsymbol{\gamma}}_{\mathbf{D}} = \left(\hat{\gamma}_{1,D},\dots,\hat{\gamma}_{n+3n_C,D}\right)'$ with $\hat{\gamma}_{i,D} = 1$ for $i = 1\dots n_U + m$; and, depending on the donor search ceasing time t_i (see lower part of Table S1),

- $\hat{\gamma}_{i,D} = \hat{S}_D(3)$, $\frac{\hat{S}_D(3)}{\hat{S}_D(0.5)}$, or $\frac{\hat{S}_D(3)}{\hat{S}_D(1)}$ for $i = n_U + m + 1\dots n$
- $\hat{\gamma}_{i,D} = 1 - \hat{S}_D(0.5)$ or zero for $i = n+1\dots n+n_C$
- $\hat{\gamma}_{i,D} = \hat{S}_D(0.5) - \hat{S}_D(1)$, $\frac{\hat{S}_D(0.5) - \hat{S}_D(1)}{\hat{S}_D(0.5)}$, or zero for $i = n+n_C+1\dots n+2n_C$, and
- $\hat{\gamma}_{i,D} = \hat{S}_D(1) - \hat{S}_D(3)$, $\frac{\hat{S}_D(1) - \hat{S}_D(3)}{\hat{S}_D(0.5)}$, or $\frac{\hat{S}_D(1) - \hat{S}_D(3)}{\hat{S}_D(1)}$ for $i = n+2n_C+1\dots n+3n_C$

We need three $n+3n_C$-dimensional vectors, $\mathbf{x}_D^{(0.5)}$, $\mathbf{x}_D^{(1)}$, and $\mathbf{x}_D^{(3)}$, to indicate group membership (Table S2).

Table S2: Components of the three group membership vectors of the second WPV model of simulation study 1. Here $\mathrm{I}(.)$ denotes the indicator function, and t_i is used to denote donor identification times.

for	$x_{i,D}^{(0.5)}$	$x_{i,D}^{(1)}$	$x_{i,D}^{(3)}$
$i = 1\dots n_U$	0	0	0
$i = n_U + 1\dots n_U + m$	$\mathrm{I}(t_i = 0.5)$	$\mathrm{I}(t_i = 1)$	$\mathrm{I}(t_i = 3)$
$i = n_U + m + 1\dots n$	0	0	0
$i = n+1\dots n+n_C$	1	0	0
$i = n+n_C+1\dots n+2n_C$	0	1	0
$i = n+2n_C+1\dots n+3n_C$	0	0	1

Now, $\bar{V}_{0,D} = \frac{\sum_{i=1}^{n+3n_C}\left(1-x_{i,D}^{(0.5)}\right)\left(1-x_{i,D}^{(1)}\right)\left(1-x_{i,D}^{(3)}\right)\hat{\gamma}_{i,D}\hat{V}_{i,D}\left(t^*\right)}{\sum_{i=1}^{n+3n_C}\left(1-x_{i,D}^{(0.5)}\right)\left(1-x_{i,D}^{(1)}\right)\left(1-x_{i,D}^{(3)}\right)\hat{\gamma}_{i,D}}$ and $\bar{V}_{1,D}^{(j)} = \frac{\sum_{i=1}^{n+3n_C} x_{i,D}^{(j)}\hat{\gamma}_{i,D}\hat{V}_{i,D}\left(t^*\right)}{\sum_{i=1}^{n+3n_C} x_{i,D}^{(j)}\hat{\gamma}_{i,D}}$ provide estimators of $S_0\left(t^*\right)$ and $S_1\left(t^*|\,j\right)$, for $j \in \{0.5,1,3\}$, respectively. Note that the estimate $\bar{V}_{0,D}$ for $S_0\left(t^*\right)$ is the same as for the first WPV model above.

Of course, the vector $\hat{\mathbf{V}}_{\mathbf{D}}$ can also be used as response variable in a weighted generalised linear model with linear predictor $\beta_{0,D} + \beta_{1,D}^{(0.5)} x_{i,D}^{(0.5)} + \beta_{1,D}^{(1)} x_{i,D}^{(1)} + \beta_{1,D}^{(3)} x_{i,D}^{(3)}$.

Remark: The predefined true survival probability values for simulation study 1 are $S_0\left(t^*\right) = 0.333$, $S_1\left(t^*\right) = 0.620$, $S_1\left(t^*|0.5\right) = 0.733$, $S_1\left(t^*|1\right) = 0.681$ and $S_1\left(t^*|3\right) = 0.451$, respectively.

Section C

Figure S1: True survival curves of patients with (dashed line) and without (solid line) available donor are shown together with the corresponding waiting time distributions (dotted line) for the seven scenarios of simulation study 2. Further details of the simulation setup are provided in Pötschger et al. [2] and its supplement (Additional file 1). The latter contains the original Figure S1 with a time range of 5 years; in the adaptation below the time range has been extended to 10 years.

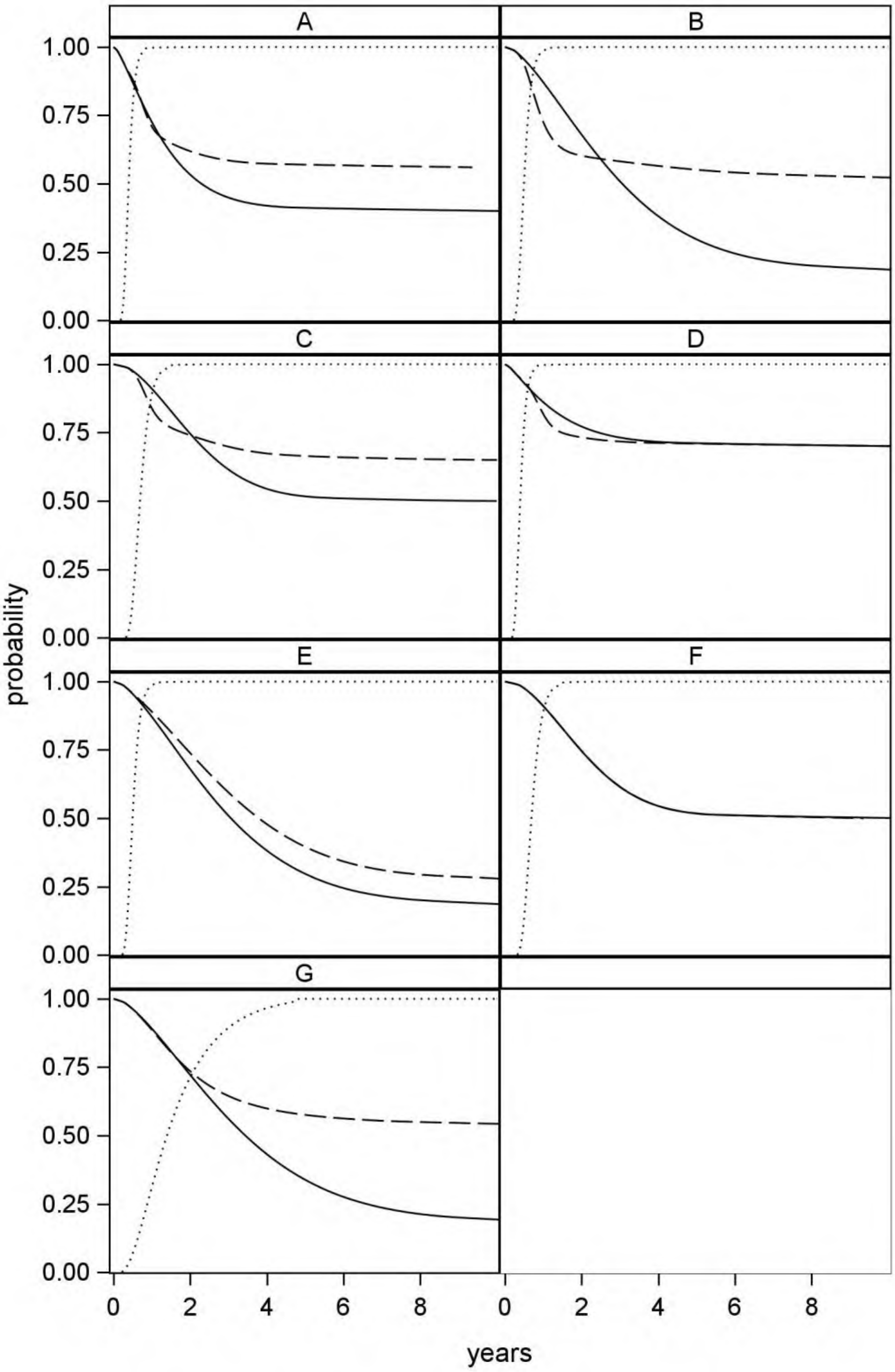

Section D: Varying the long-time survival time $t*$ and the proportion of available donors in Simulation Study 2

Note that simulation results of the main manuscript have not been included in the following; instead, new computations have been performed. That is, results of Figures S2 and S3 may slightly differ from corresponding results of Figure 4 as different starting seeds for the random number generator have been used. The same applies to results of Figures S8 and S9 that correspond to results of Figure 5.

Figure S2: Bias distribution of log-minus-log survival probabilities and log-cumulative hazard ratios estimated with both the GPV and the WPV approach in simulation study 2 (1000 repetitions) for a sample size of $n = 400$. The outcome is evaluated at $t* = 5$ for each of the seven scenarios A-G (see Figure S1 in Section C). The maximum donor search time t^{search} is set to 5 years throughout. Always six boxplots are grouped together; they correspond to proportions of available donors of 0.2, 0.3, 0.4, 0.5, 0.6, 0.7 for scenarios A-F, and 0.25, 0.35, 0.45, 0.55, 0.65, 0.75 for scenario G, respectively.

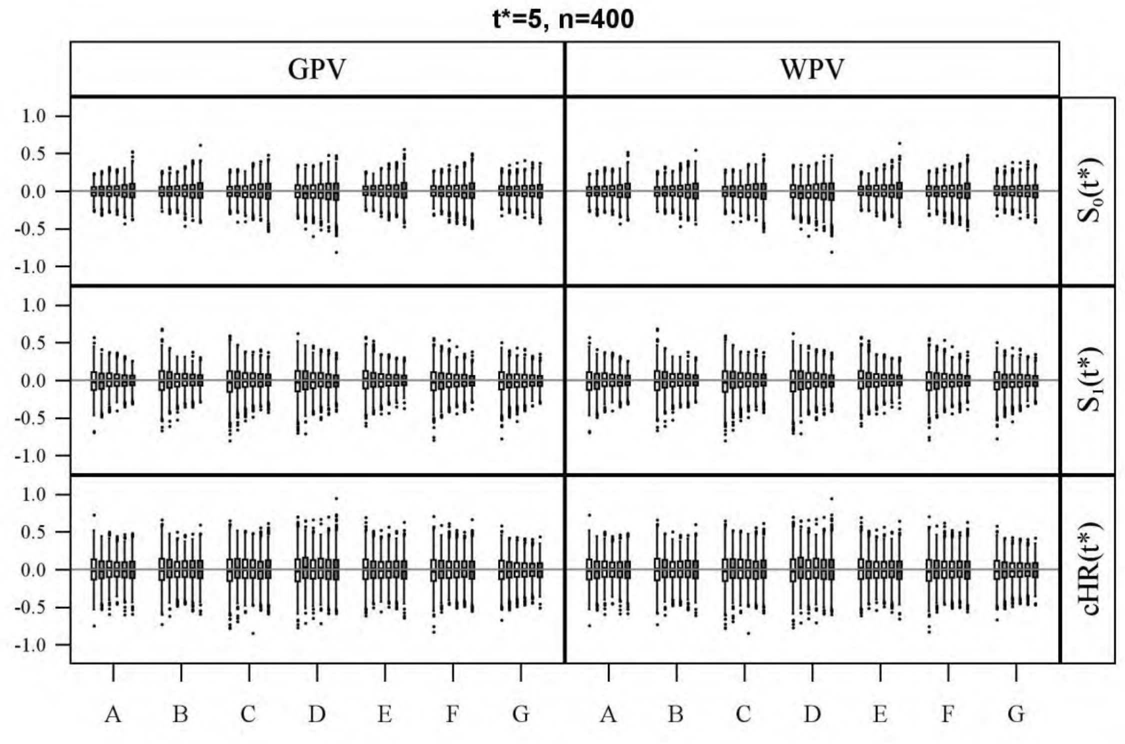

Figure S3: Bias distribution; the setting of Figure S2 is repeated with $t^* = 5$ and $n = 1000$.

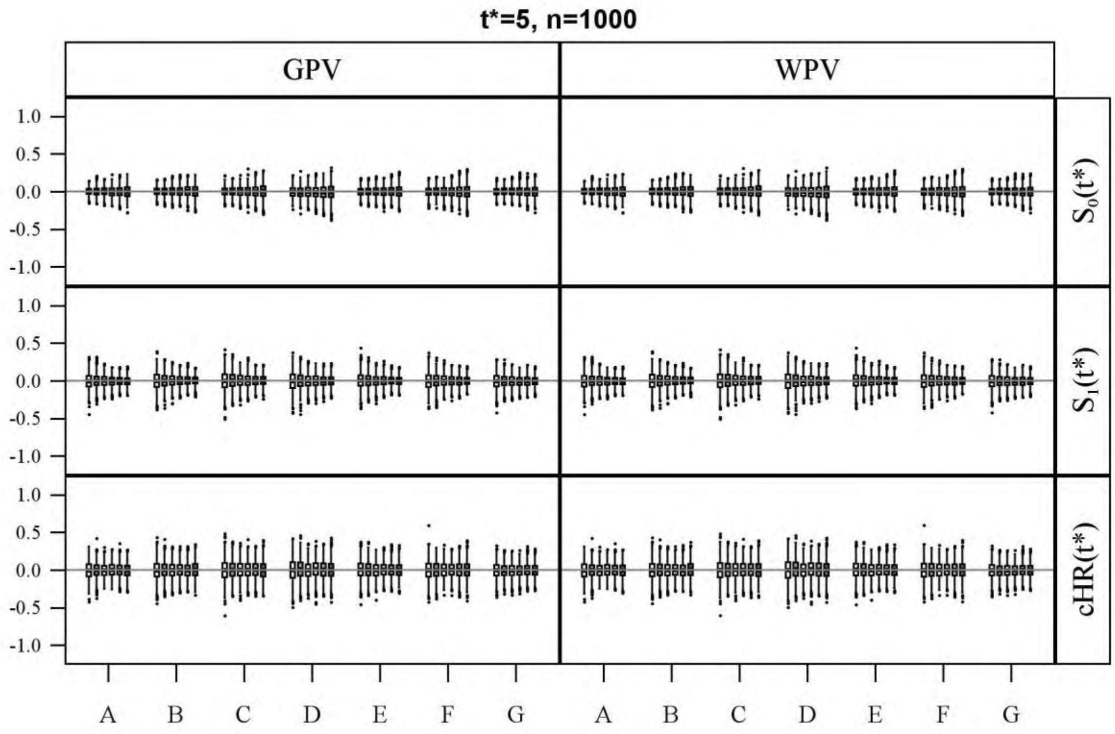

Figure S4: Bias distribution; the setting of Figure S2 is repeated with $t^* = 7$ and $n = 400$.

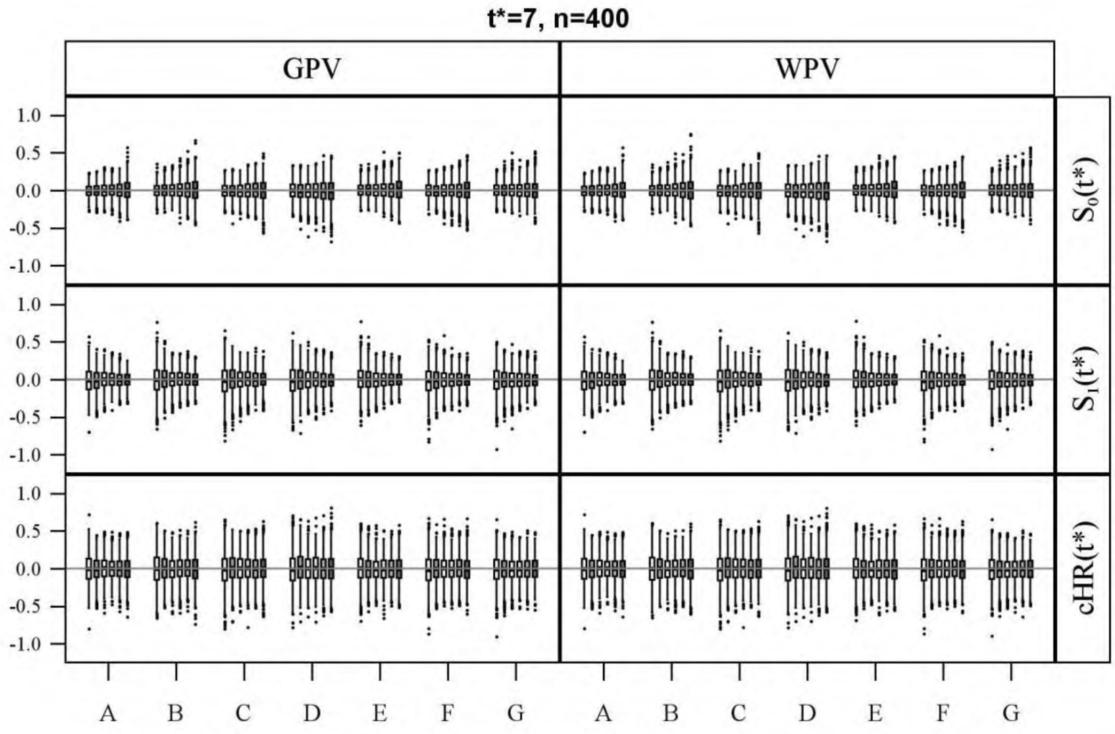

Figure S5: Bias distribution; the setting of Figure S2 is repeated with $t^* = 7$ and $n = 1000$.

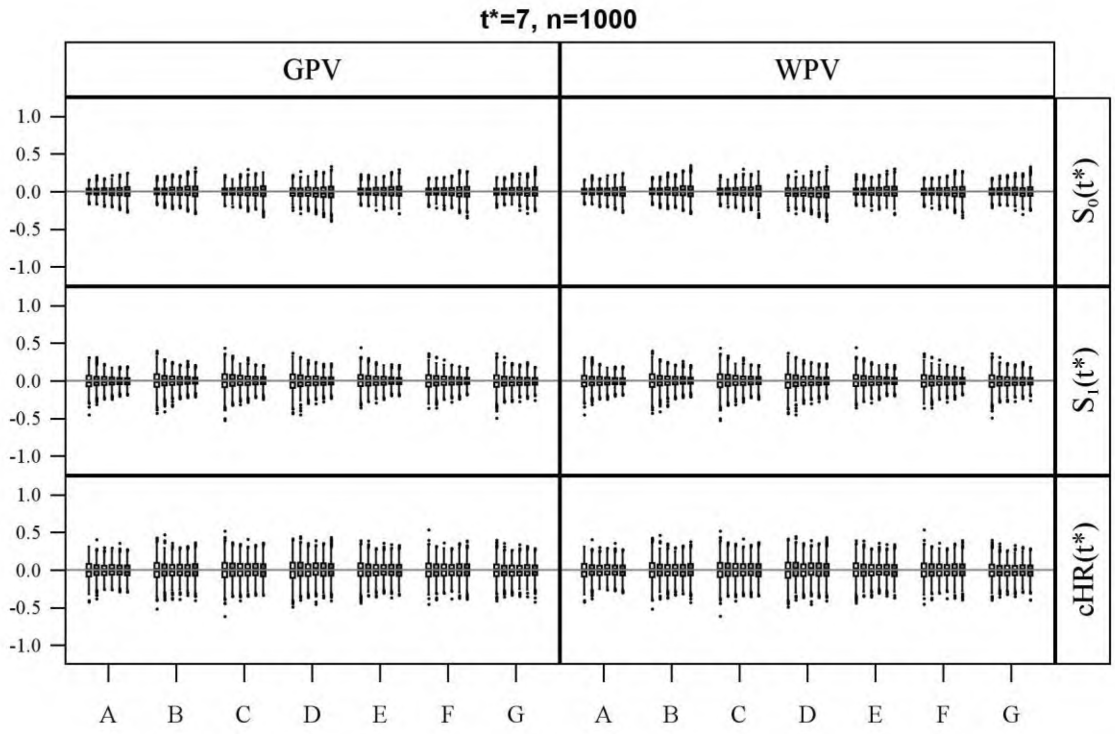

Figure S6: Bias distribution; the setting of Figure S2 is repeated with $t^* = 9$ and $n = 400$. See also Table S3 below.

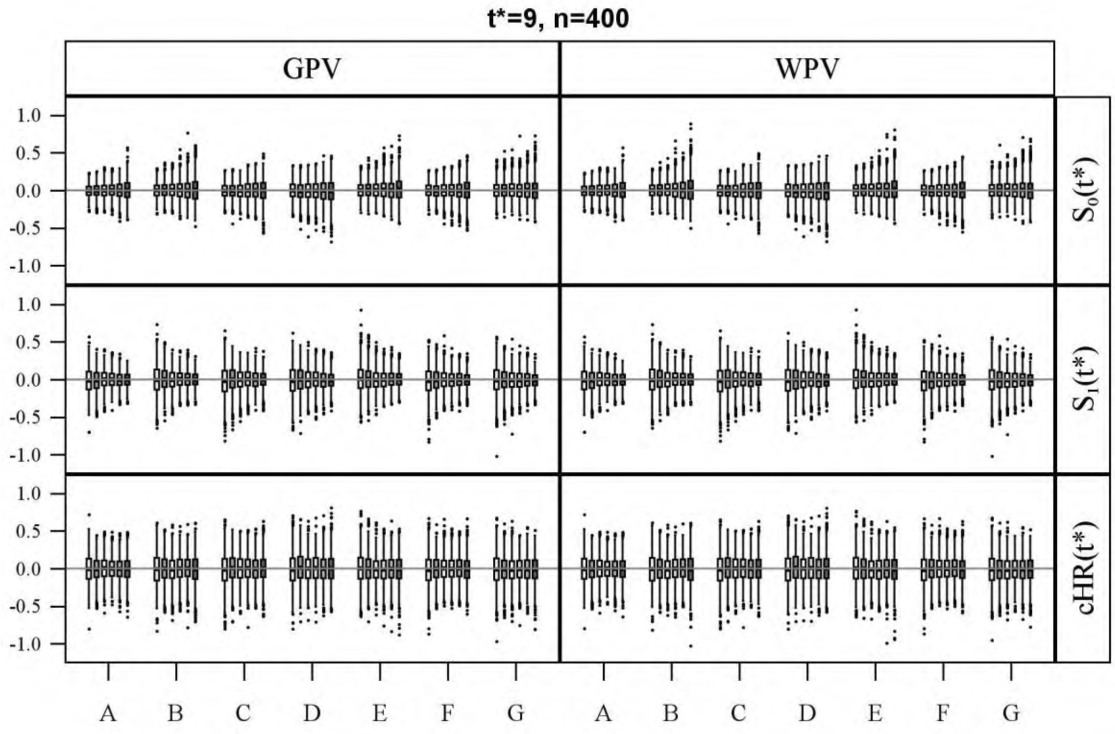

Figure S7: Bias distribution; the setting of Figure S2 is repeated with $t^*=9$ and $n=1000$.

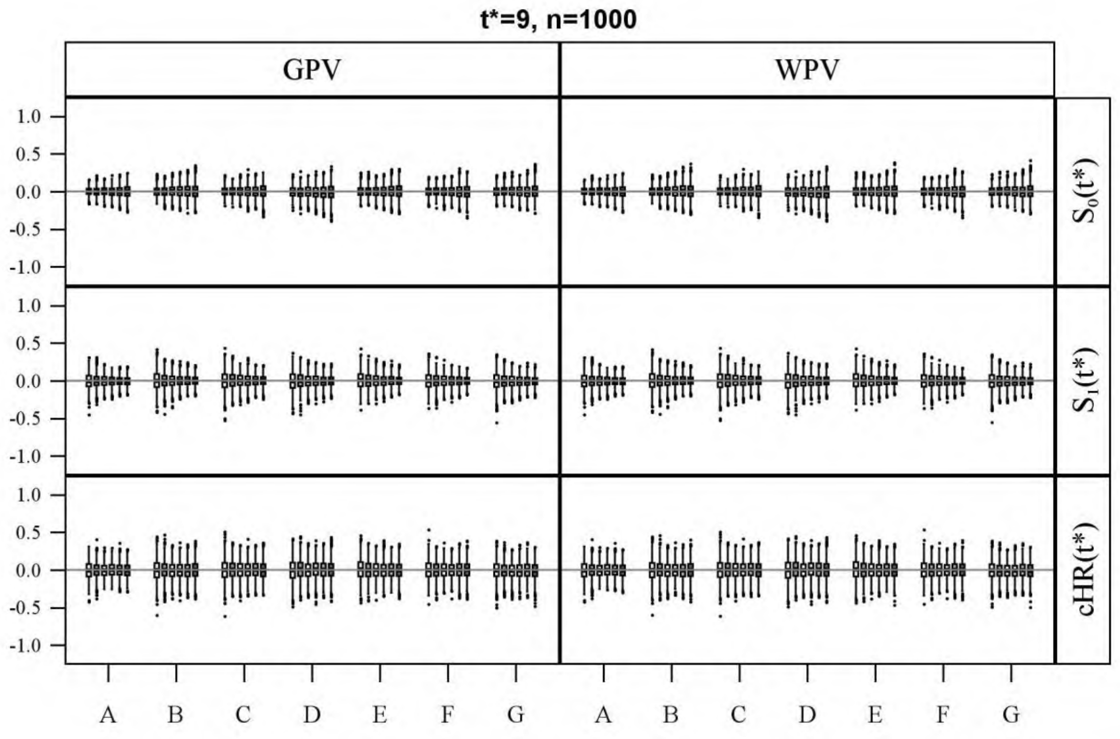

Figure S8: Observed coverage rates of 95 % confidence intervals for survival probabilities and cumulative hazard ratios estimated with both the GPV and the WPV approach in simulation study 2 (1000 repetitions) for a sample size of $n = 400$. The outcome is evaluated at $t^* = 5$ for each of the seven scenarios A-G (see Figure S1 in Section C). The maximum donor search time t^{search} is set to 5 years throughout. The following six proportions of available donors are studied for scenarios A-F: 0.2 (circle), 0.3 (circle-filled), 0.4 (triangle), 0.5 (triangle-filled), 0.6 (square), and 0.7 (square-filled). The slightly different donor proportions 0.25, 0.35, 0.45, 0.55, 0.65, and 0.75 are used for scenario G.

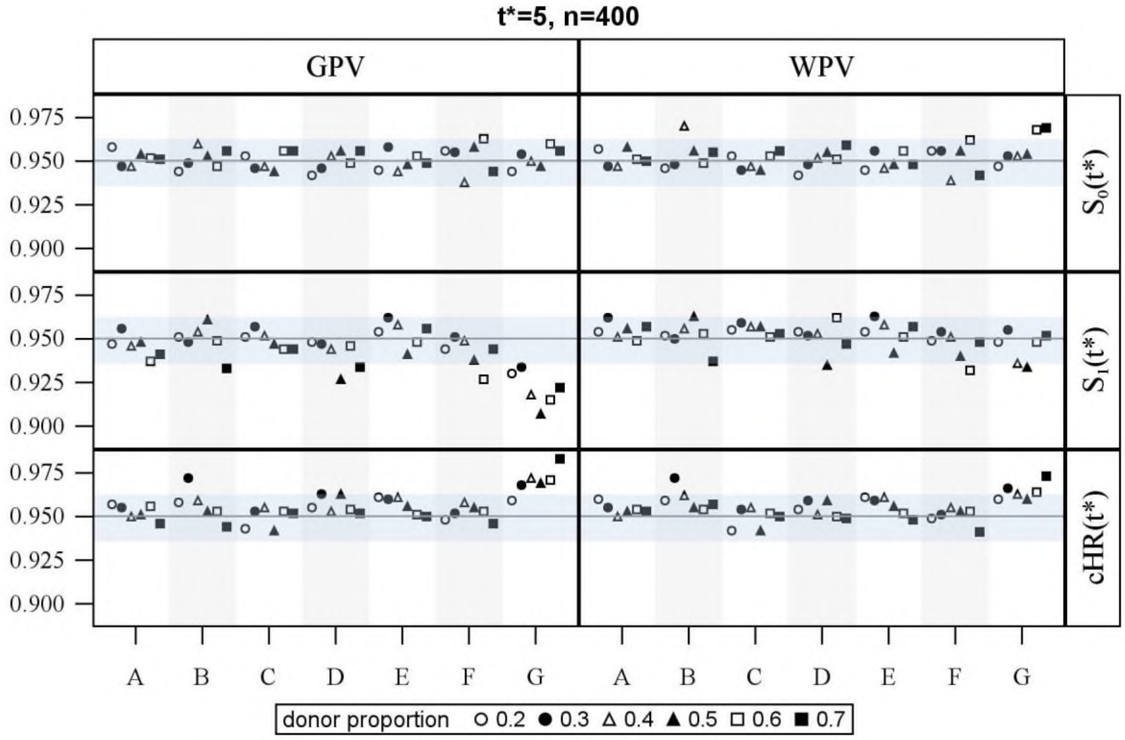

Figure S9: Observed coverage rates of 95 % confidence intervals; the setting of Figure S8 is repeated with $t^* = 5$ and $n = 1000$.

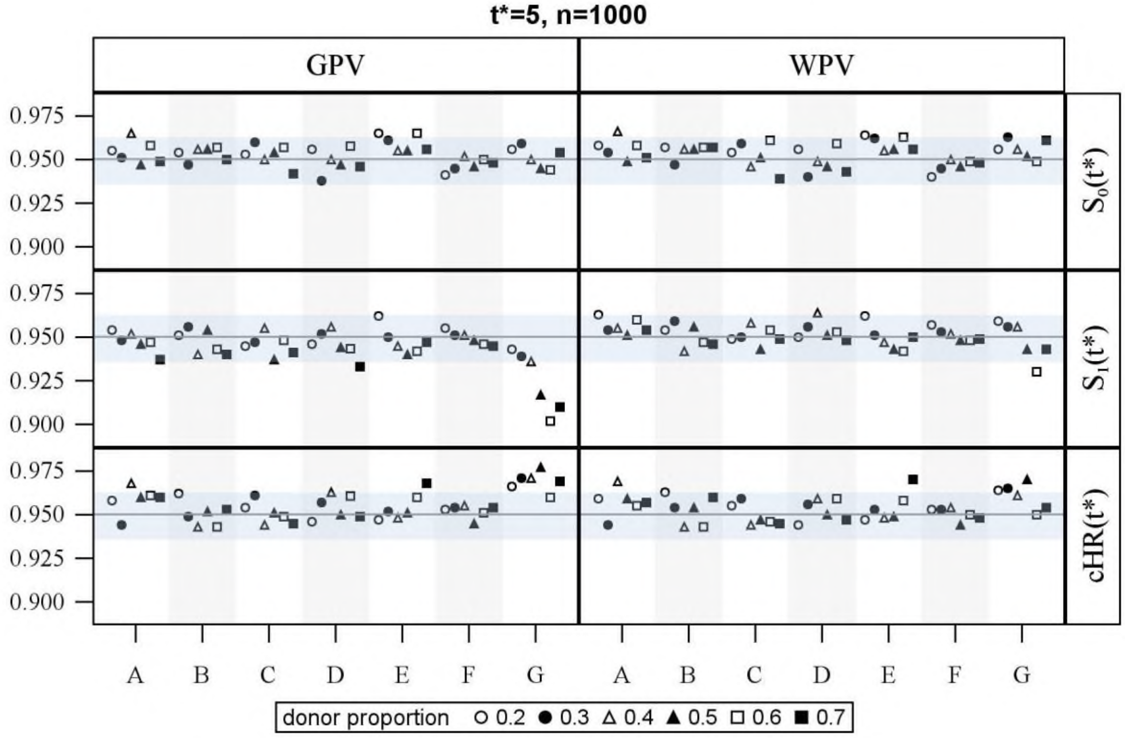

Figure S10: Observed coverage rates of 95 % confidence intervals; the setting of Figure S8 is repeated with $t^* = 7$ and $n = 400$.

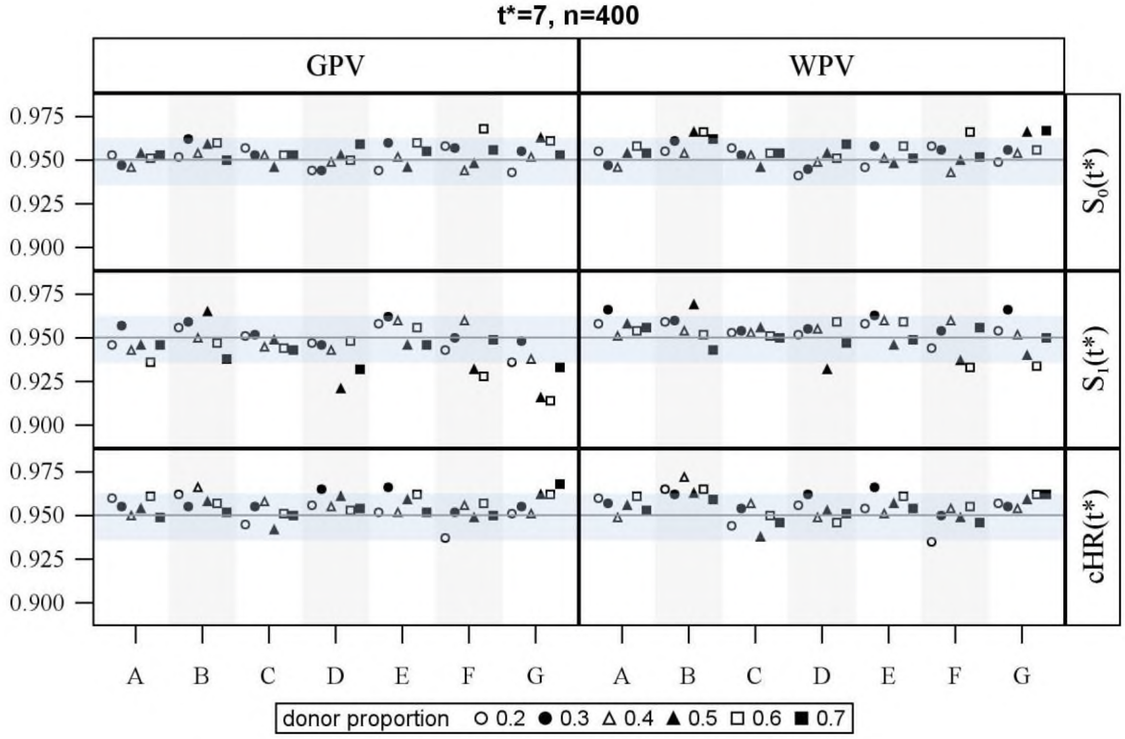

Figure S11: Observed coverage rates of 95 % confidence intervals; the setting of Figure S8 is repeated with $t^* = 7$ and $n = 1000$.

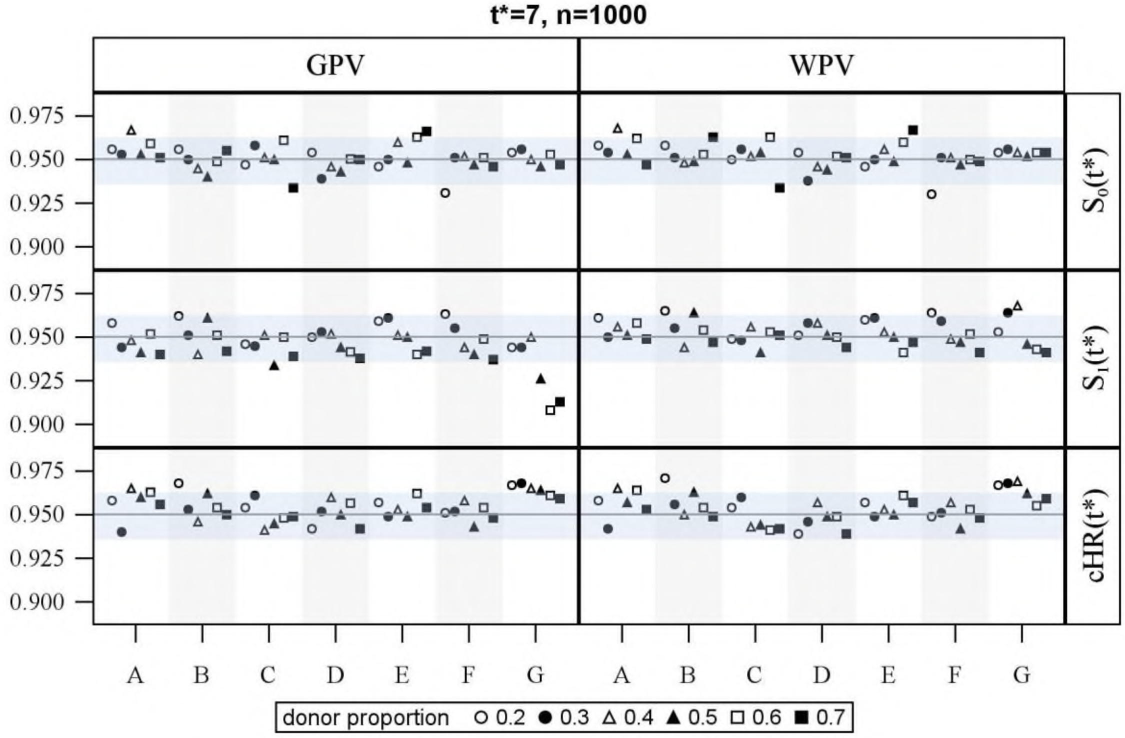

Figure S12: Observed coverage rates of 95 % confidence intervals; the setting of Figure S8 is repeated with $t^* = 9$ and $n = 400$. See also Table S3 below.

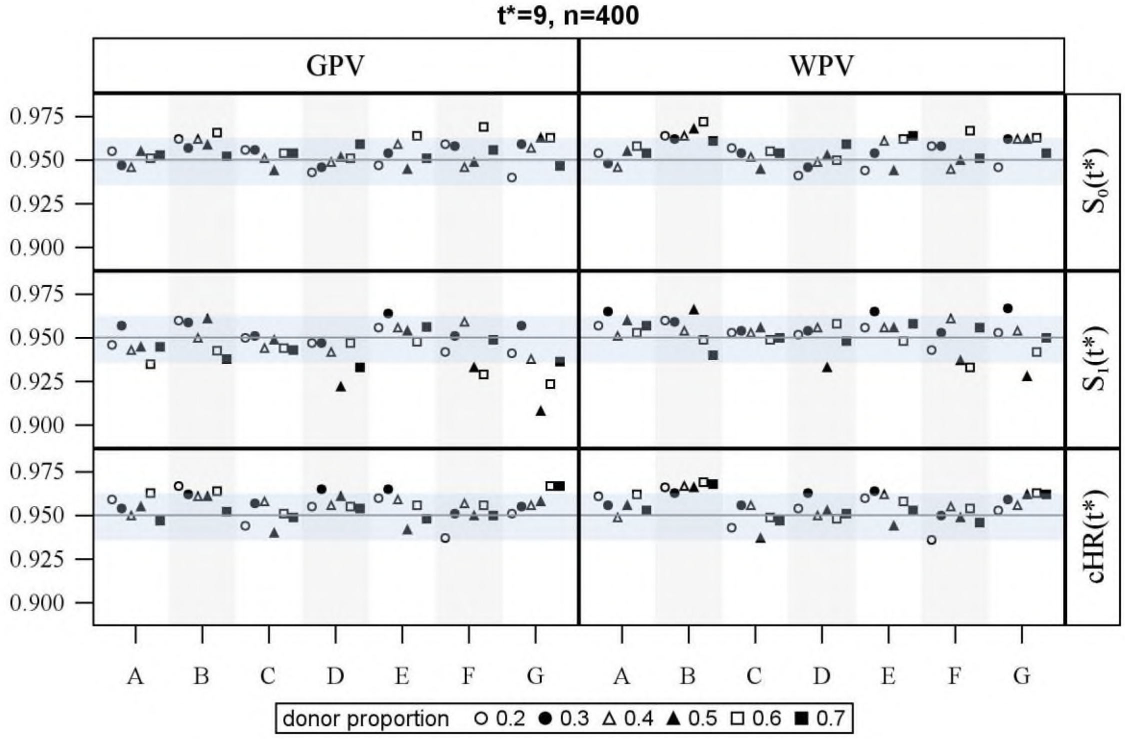

Figure S13: Observed coverage rates of 95 % confidence intervals; the setting of Figure S8 is repeated with $t^* = 9$ and $n = 1000$.

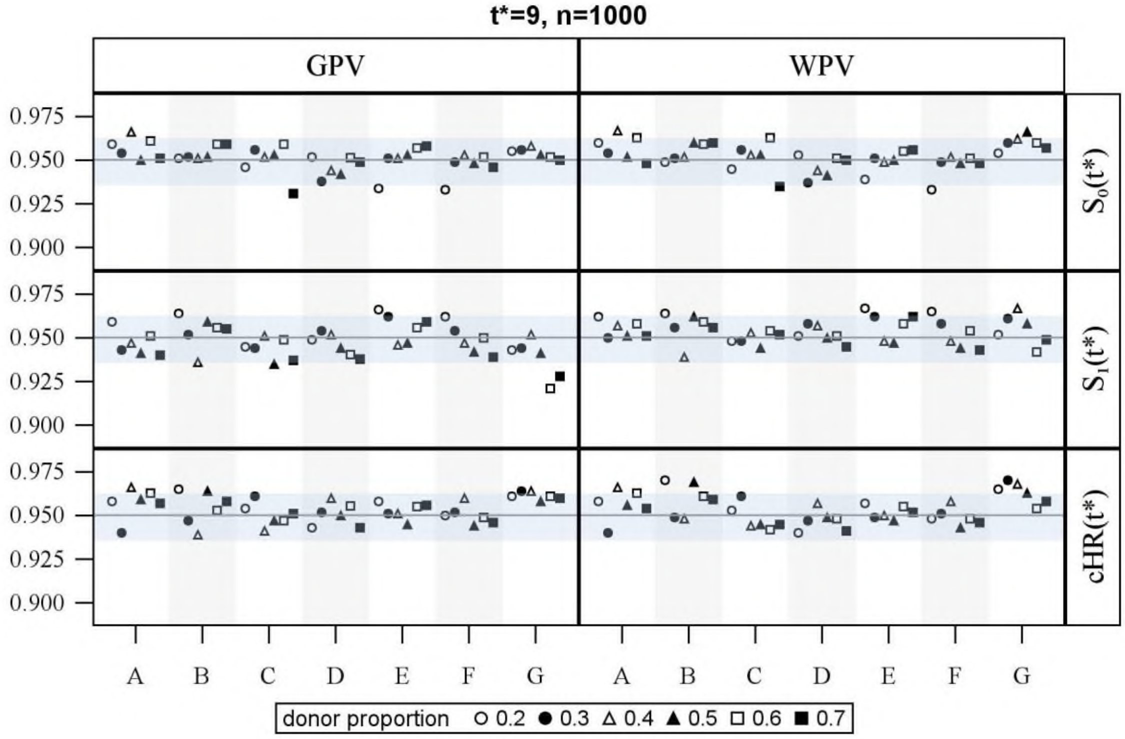

Table S3: For $n = 400$ and $t^* = 9$, some of the results shown in Figures S6 and S12 are based on less than 1000 simulation runs. In this extreme setting, the weighted generalised linear model could not always provide estimates (mostly for the GPV approach alone, only once for both approaches together).

Approach	Scenario	Proportion of available donors	Simulation runs without results
GPV	B	0.3	1
GPV	B	0.5	2
GPV	B	0.6	4
GPV	B	0.7	20
both	E	0.2	1
GPV	E	0.5	1
GPV	E	0.6	4
GPV	E	0.7	17
GPV	G	0.65	6
GPV	G	0.75	8

Section E: Comparing the computing time of the GPV and the WPV approach

Figure S14: For simulation study 2, the seven scenarios (A-G), the two sample sizes (400 and 1000), the three $t*$ values (5, 7, and 9 years), and the six proportions of available donors (0.2, 0.3, 0.4, 0.5, 0.6, 0.7 for scenarios A-F, and 0.25, 0.35, 0.45, 0.55, 0.65, 0.75 for scenario G) were combined. Ten simulation data sets were generated for each of these 252 combinations and the corresponding runtimes of SAS 9.4 were measured for both the GPV and the WPV approach at our local computer hardware setting. For each combination, the ratio of the median runtime of the GPV to the median runtime of WPV approach was computed.

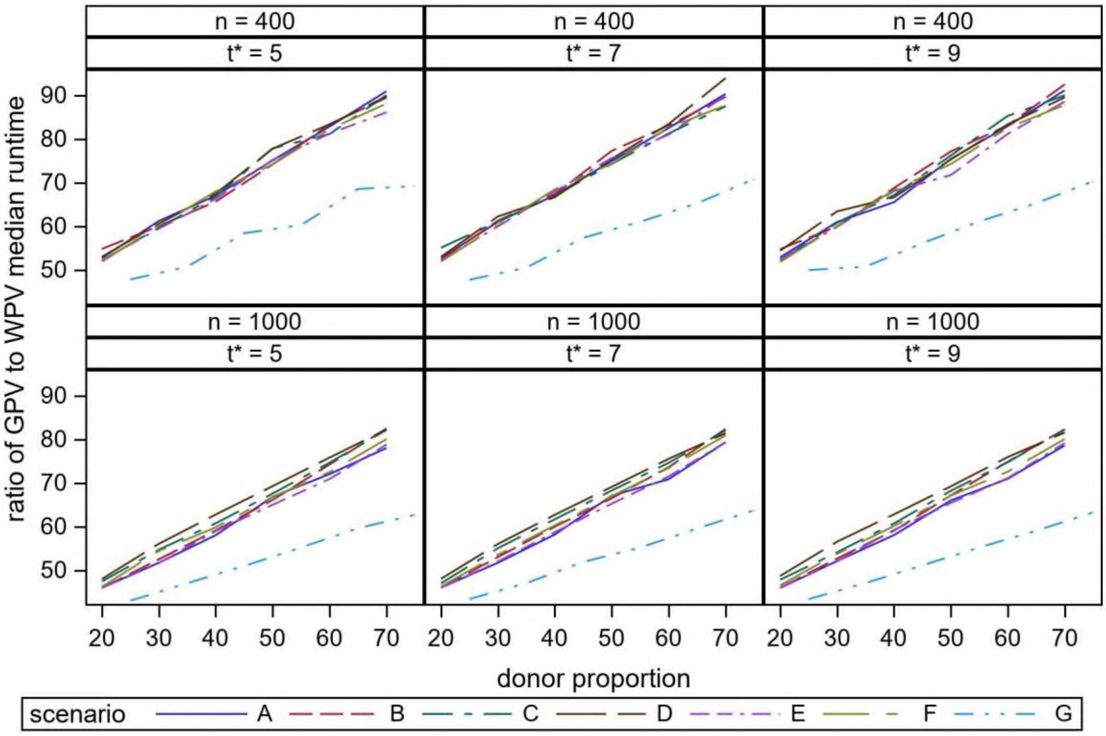

References

1. Sposto R. Cure model analysis in cancer: an application to data from the Children's Cancer Group. *Statistics in Medicine* 2002; 21: 293-312. DOI: 10.1002/sim.987.
2. Pötschger U, Heinzl H, Valsecchi MG, et al. Assessing the effect of a partly unobserved, exogenous, binary time-dependent covariate on survival probabilities using generalised pseudo-values. *BMC Medical Research Methodology* 2018; 18: 14. DOI: 10.1186/s12874-017-0430-5.
